# A genetic algorithm based method for stringent haplotyping of family data

**DOI:** 10.1186/1471-2156-10-57

**Published:** 2009-09-17

**Authors:** Francois Besnier, Örjan Carlborg

**Affiliations:** 1 Linnaeus Centre for Bioinformatics, Uppsala University, SE-75124 Uppsala, Sweden; 2 Department of Animal Breeding and Genetics, Swedish University of Agricultural Sciences SE-750 07 Uppsala Sweden

## Abstract

**Background:**

The linkage phase, or haplotype, is an extra level of information that in addition to genotype and pedigree can be useful for reconstructing the inheritance pattern of the alleles in a pedigree, and computing for example Identity By Descent probabilities. If a haplotype is provided, the precision of estimated IBD probabilities increases, as long as the haplotype is estimated without errors. It is therefore important to only use haplotypes that are strongly supported by the available data for IBD estimation, to avoid introducing new errors due to erroneous linkage phases.

**Results:**

We propose a genetic algorithm based method for haplotype estimation in family data that includes a stringency parameter. This allows the user to decide the error tolerance level when inferring parental origin of the alleles. This is a novel feature compared to existing methods for haplotype estimation. We show that using a high stringency produces haplotype data with few errors, whereas a low stringency provides haplotype estimates in most situations, but with an increased number of errors.

**Conclusion:**

By including a stringency criterion in our haplotyping method, the user is able to maintain the error rate at a suitable level for the particular study; one can select anything from haplotyped data with very small proportion of errors and a higher proportion of non-inferred haplotypes, to data with phase estimates for every marker, when haplotype errors are tolerable. Giving this choice makes the method more flexible and useful in a wide range of applications as it is able to fulfil different requirements regarding the tolerance for haplotype errors, or uncertain marker-phases.

## Background

The average number of recombinations between two linked markers is a function of the distance between them [1,2]. Therefore, recombination events are rare between closely linked markers, and the alleles are transmitted as large haplotype blocks from parents to offspring. When tracing inheritance of alleles in multi-generation pedigrees, haplotype data is more informative that raw genotype data. This is utilized by e.g. Meuwissen and Goddard (2000) [3] to infer the co-variance of base generation individuals (i.e. founder individuals without known parents in the pedigree), at a given locus in their IBD based fine mapping strategy.

As large-scale genotyping of Single Nucleotide Polymorphism (SNP) markers is now affordable, the density of marker maps used in genome studies for dissection of complex traits will increase, whereas very dense maps are already used in association studies and genomic selection. The information content of each bi-allelic SNP marker is, however, often low in studies of outbred populations. Therefore, they are usually grouped by haplotype segments containing *n *SNP markers [4,5]. As shown by Grapes et al (2005) [4] the value of *n *can be optimized to maximize the accuracy of the IBD prediction as well as the resolution of the fine mapping.

As commonly used genotyping techniques do not provide haplotype information, several algorithms have been developed to estimate haplotypes from genotype data such as: *Deterministic *or *rule based *reconstruction based on *Minimum Recombinant Haplotype Configuration *(MRHC) [6,7], *Stochastic *reconstruction based on either *Markov chain descent graph *[8], or other *Monte Carlo *approaches [9-11], *Descent tree likelihood *[12], *Maximum likelihood *by an EM algorithm [13,14] or MRHC combined with a Genetic Algorithm (GA) [15]. Some algorithms reconstruct haplotypes using both genotype and pedigree information [6-8,14] whereas other methods use only the genotype. In the present study, we focus on haplotype reconstruction in family data where both genotype and pedigree information are available.

The haplotypes inferred with any of the methods currently available will unavoidably contain errors [16], and most methods do not provide information about how certain the haplotype estimates are. Sometimes the most likely haplotype configuration is reported together with an estimate of the haplotyping error rate obtained by means of simulation [7]. The error risk related to each ordered marker is however still unknown. Others [12] allow the user to distinguish between ordered and uncertain genotypes, even though the criterion used to differentiate resolved from unresolved cases is not accessible to the user. In Becker and Knapp's approach [14], a likelihood weight can be used to evaluate the error risk of the complete inferred haplotypes, but no assessment is available for individual ordered loci.

Here, we describe a method to infer haplotypes that provides an estimate of every ordered locus, with the novelty that the user can select the stringency criterion used to decide the maximum error risk acceptable for each marker.

To make haplotype information useful for linkage mapping in deep pedigrees, the haplotype errors rate needs to be controlled or at least known. This as a single erroneous haplotype in the first generations of a deep and complex pedigree will propagate and lead to significant errors in the estimated structure of IBDs. As most existing methods for haplotype estimation neither allow the user to control the error rate nor provide estimates of the error risk associated with individual inferred markers, they are not adapted for use in this application. Here, we describe a new Genetic Algorithm-based haplotyping method that includes a stringency criterion that allows the user to control the haplotype error rate by discarding ordered genotypes associated with higher risk of error.

## Methods

### Genetic Algorithm

Genetic Algorithms (GAs) are iterative procedures implemented in computer programs with the aim of solving optimization problems. A set of possible solutions (called individuals or haplotypes) is sampled from an original population, and evaluated for their fitness. Solutions are then chosen at a frequency proportional to their fitness and modified (mutation, recombination) to form a new population that will be used in the next iteration of the algorithm. We have implemented a GA based algorithm for haploptype reconstruction [15], where the GA was used to identify the most likely set of ordered genotypes among all possible ones in a given individual. Each ordered genotype provides the parental origin of the alleles, with for example maternally inherited allele noted first, and paternally inherited noted second. Global optimisation algorithm approaches are needed to reconstruct ordered genotypes, as large numbers of individuals and genotyped loci in a data set makes the exploration of all possible haplotypes computationally intractable. The GA thus replaces the exhaustive search over all possible configurations and instead, uses iterative sampling and evaluation of potential haplotypes until it converges towards an optimum configuration.

### Haplotype inference

Haplotypes are inferred using a four step recursive approach, where step one and two were adapted from Qian and Beckmann [6]. The algorithm successively i) infers the parental origin of the alleles of the progeny when they can be deduced with certainty from the parental genotype(s), ii) infers missing genotype(s) from the genotype and haplotype of both parents and offspring of the individual, iii) infers the haplotype of the parents based on the ordered genotype of the offspring using a genetic algorithm, and iv) infers the most likely haplotype of the progeny based on parental haplotype information using a genetic algorithm.

***Step 1 ***- inference of unambiguous parental origins of offspring alleles using parental genotypes:

The parental origin of all offspring alleles in the pedigree is reconstructed when it can be unambiguously inferred from the parental genotype (e.g. when both two parents are homozygotes). An exhaustive list of possible cases can be found in Quian and Beckmann (2002) [6].

***Step 2 ***- inference of missing marker genotypes from the nearest relative information:

Missing marker genotypes due to for instance sample loss or technical problems in the genotyping procedure are inferred. In practice, the missing data can often be deduced using genotype and haplotype information of its nearest relatives (i.e. parents and offspring). The algorithm used to infer missing genotypes utilizes full-sib family data: let the two alleles of an offspring be m_1 _and m_2_, the two alleles of the sire ms_1 _and ms_2_, and the two alleles of the dam md_1 _and md_2_. The parental origin of the offspring alleles is P_mo_. If P_mo _is known, m_1 _and m_2 _are the paternally and maternally inherited alleles in the offspring genotype respectively. The following rules are used to determine the missing genotype from the marker genotype of the parents:

-if m_1 _is missing and ms_1 _= ms_2 _and m_2 _≠ ms_1_, then m_1 _= ms_1_

-if m_2 _is missing and md_1 _= md_2 _and m_1 _≠ md_1_, then m_2 _= md_1_

Or from the ordered genotype:

-if ms_1 _is missing and P_mo _is known and ms_2 _≠ m_1 _then ms_1 _= m_1_

-if ms_2 _is missing and P_mo _is known and ms_1 _≠ m_1 _then ms_2 _= m_1_

-if md_1 _is missing and P_mo _is known and md_2 _≠ m_2 _then md_1 _= m_2_

-if md_2 _is missing and P_mo _is known and md_1 _≠ m_2 _then md_2 _= m_2_

These rules are applied to every individual in the pedigree before proceeding to step 3.

***Step 3 ***- inference of haplotypes of parents using offspring haplotypes

A Genetic Algorithm is used to infer haplotypes of parents using the ordered genotype of their offspring. For each parent, a binary GA chromosome of given length *l *is created, where *l *is the number of non-ordered genotypes in the parent. Each variable in the GA-chromosome represents a potential set of ordered genotypes, and each of its elements takes the value 0 or 1 representing paternal or maternal origin of the two marker alleles in the un-ordered genotype. The evaluation function for each sampled GA-chromosome is the likelihood of the recombination events that are needed in the parental gametes to generate the observed haplotypes of all offspring of the individual: The Likelihood is computed as a function of the recombination probability between the markers

(1)

where *n *is the number of offspring for a given parent and *m *the number of intervals between the markers we try to resolve. P(r_ij_) is the probability to observe a recombination in the parental gamete of offspring *i *in marker interval *j*.

**Figure 1:**
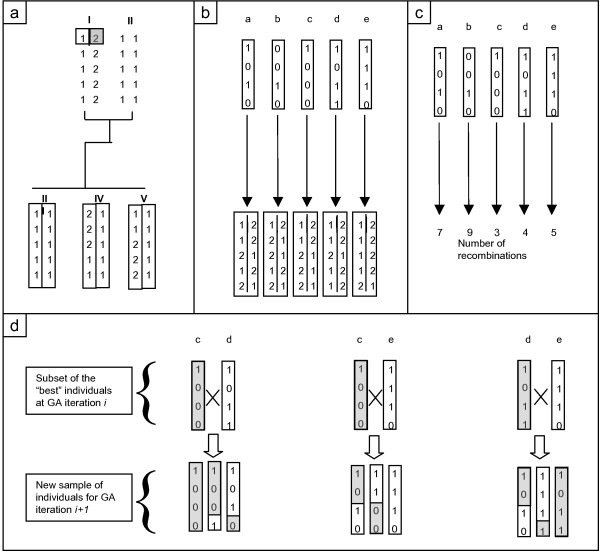
**Implementation of the genetic algorithm**. a) In a full sib family, the haplotype of individual I is to be estimated. Since no parent is available, the first heterozygous marker is phased arbitrarily allele "1" being of paternal origin and allele "2" being of maternal origin. The three offspring have been phased by a deterministic approach. b) Step1: Five GA individuals are randomly sampled (a, b, c, d, e). Each sample is a possible haplotype of the non-phased markers of individual I. c) Step2: Fitness of the GA individuals is computed as a likelihood function of the number of recombinations observed in the offspring chromosomes. d) Step3: The GA individuals with higher fitness (c, d, e) are selected and crossed in a simulated diploid reproduction mechanism that generates the sample of GA individuals for the next generation.

In our implementation we use a Genetic Algorithm from in the library PGAPack [17]. An overview of the procedure for our GA is given in Figure 1.

To determine the haplotype of an individual, a set of haplotype configurations is randomly sampled (Step 1, Figure 1). The relative fitness of each haplotype is evaluated as the likelihood of the needed recombination events, given the estimated linkage map in the population. In our implementation, the likelihood of a sampled haplotype depends on the likelihood of the recombination events observed in the offspring of the individual, given that the sampled haplotype is the true parental one (Step 2, Figure 1). The next step of the GA is to generate a new set of haplotypes to be evaluated using the known fitness of previous evaluations (Step 3, Figure 1). Here, haplotypes are sampled at frequencies proportional to their fitness.

To determine the risk of error associated with each ordered genotype, we use the fact that the GA is a stochastic global optimization algorithm with relatively poor local convergence. Consequently, when multiple "best" haplotypes with similar fitness exist, the GA will converge to different optima in different runs. We thus iterate step 3 *k *times for each parent in a given generation (*k *is defined by the user) and calculate the proportion of the *k *iterations that converge to the globally best solution. If this proportion is high it provides a strong indication that there is a single dominating optimum in the parameter space. When it is low, it indicates the existence of multiple and almost equally likely optima, and that consequently the ability to discriminate between true and false haplotypes is low.

***Step 4 ***- inference of ambiguous genotype order in the offspring

After inferring the haplotype of the parents in a given generation (*g*) in step 3, we use the genetic algorithm to infer the ordered genotypes of the individuals in generation (g+1) that could not be inferred in step1. For each offspring, we use a binary GA of length *l*, where *l *is the number of unordered genotypes in the offspring. For each sampled haplotype configuration, the evaluation function is the likelihood of the recombination events needed in the parental gametes to generate the proposed GA haplotypes of the offspring. The Likelihood is computed as a function of the recombination probability between the markers. To estimate the error risk of each inferred haplotype, step 4 is iterated *k *times in analogy with the procedure described in step 3.

### Computational strategy for deep pedigrees

**Figure 2:**
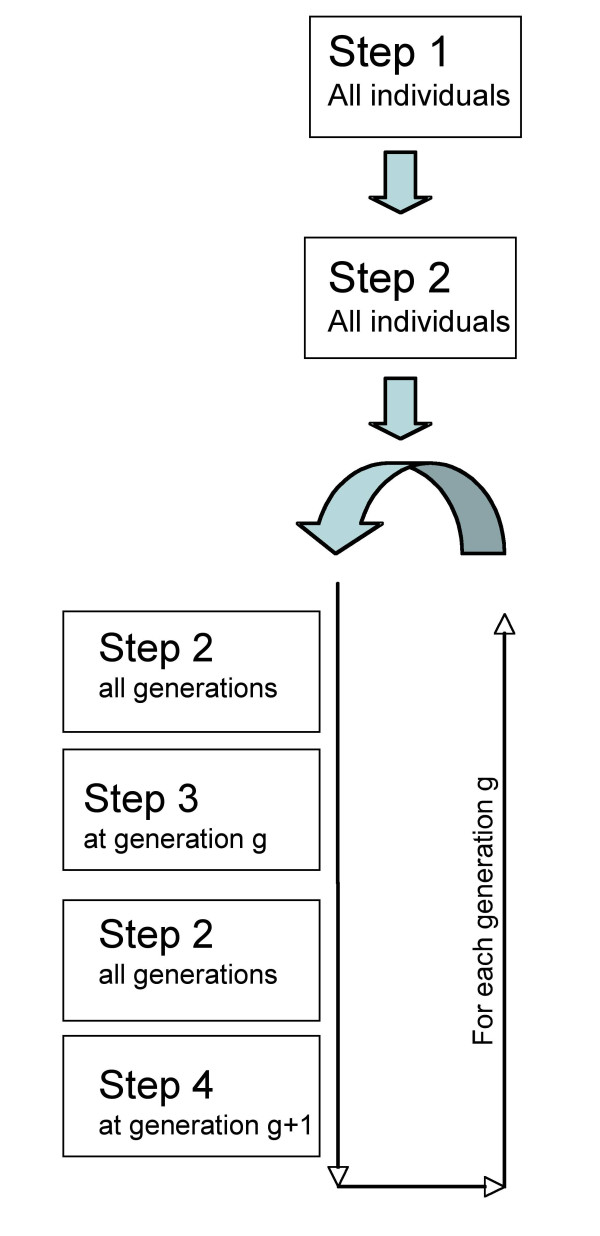
**Computational strategy for deep pedigree**. Successive steps used to estimate haplotypes in any n-generational complex pedigree.

As the algorithm is designed to infer haplotypes in multi-generational complex pedigrees, it is important to infer haplotypes one generation at a time and to apply the algorithm to the generations in an appropriate order. This way, it is possible to use the inferred information from generation *i *to infer haplotypes in generation *i*+1. Our algorithm performs the four steps described above successively for all individuals in a given generation before proceeding to the next generation (Figure 2).

**Figure 3:**
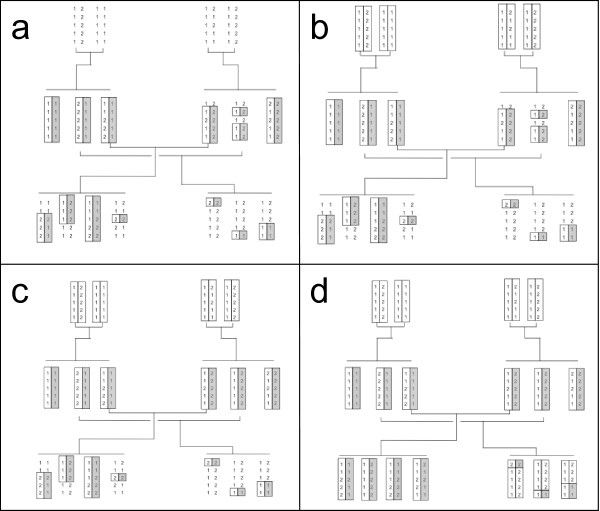
**Description of the algorithm used for estimation of haplotypes in a three generations pedigree**. Haplotypes are indicated by boxes; grey box contain maternally inherited alleles when marker phase is known, whereas two white boxes contain haplotype segments with unknown phase origin. -a haplotype estimated after the first deterministic (step 1). -b haplotype estimated in generation 1 from the offspring (step 3). -c haplotype estimated in generation 2 from their parents (step 4). -d haplotype estimated in generation 3 from their parents (step 4).

The iterative process is illustrated in Figure 3 using a simple example of a three generation pedigree. Deterministic steps 1 and 2 produce the haplotype data in Figure 3a. The genetic algorithm is then iterated for each generation separately. When inferring the haplotypes of the F_0 _generation based on the F_1_, one sets the haplotype data represented in Figure 3b. When inferring haplotypes of the F_1 _generation based on the F_0_, one sets the results in Figure 3c. Note that in this example, no additional information is gained by inferring the F_1 _generation using the F_2 _individuals. Finally, inferring the F_2 _generation using the F_1 _data gives the results shown in Figure 3d.

### Stringency criteria

Our method uses a deterministic procedure to infer unambiguous marker phases and missing genotypes, and a stochastic genetic algorithm (GA) to infer the most likely chromosome haplotype for the remaining markers. The stochastic part of the algorithm (that includes the genetic algorithm) runs *k *times for each individual chromosome (*k *determined by the user). For each chromosome in each individual, one will obtain a set of *k *ordered genotypes that have been determined in each of the *k *GA runs to be the most likely one. We now introduce a stringency criterion to be selected by the user. This criterion is the minimum frequency required for a given ordered genotype among the *k *iterations to be considered as known. Hence, if for example the stringency criterion is 0.9, an ordered genotype must be detected as the optimal solution in at least 90% of the *k *haplotypes. If the frequency of the most likely ordered genotype is smaller than 0.9, then the locus will be considered as unresolved.

The output of the algorithm thus consists of the genotype at each marker together with an indicator variable specifying if the genotype is ordered or not. If a given genotype at a marker meets the stringency criteria but is not present in all of the *k *haplotype pairs, we can replace the indicator variable with the frequency of the most likely ordered genotype for the given marker.

### Evaluation of the algorithm using simulations

The algorithm has been tested on a publicly available dataset from the 12^th ^QTLMAS Workshop [18]. This dataset has a four-generation pedigree with SNP marker information given at 0.1 cM intervals. The mating system was kept constant over generations with one male mated to 10 females. The F_0 _generation consisted of 165 individuals and the following ones 1500 individuals each. From this dataset, we selected a subset of 50 consecutive markers on the first chromosome. The inferred haplotypes and the true simulated ones were directly compared, as the true haplotype of each individual was provided in the original data set.

The algorithm was also evaluated in an experimental dataset from a nine-generation chicken advanced intercross line (AIL). The AIL founder generation (F_0_) consists of 50 individuals, generations F_1_, F_2_, F_4_, F_5_, F_6 _and F_7 _approximately 100 individuals and generations F_3 _and F_8 _contains approximately 300 and 400 individuals respectively. All individuals were genotyped with SNP markers at approximately 1 cM intervals in 13 chromosome regions. We tested the haplotyping algorithm on the largest region: a segment on chromosome 7 containing 88 markers. As the true haplotypes are not known in this experimental dataset, we used simulations to generate multiple datasets based on the same pedigree. The genotypes in the first generation were kept identical to the ones in the experimental data, whereas the genotypes in the following generations were simulated by gene dropping, taking into account the pedigree structure and marker distances of the original data set. Therefore, the genotypes in the founder generation of the simulated data were identical to the ones in the experimental population, whereas the genotypes of the following generations were sampled from the potential gametes of the founder individuals. Recombination was simulated at a rate proportional to the linkage distance between the markers. The mating pattern was kept identical to the original pedigree.

To test the algorithm, we emulate problems that are frequent in experimental data sets, and we therefore included missing genotypes in the simulated data. To explore the effect of the stringency criterion on the performance of our method, we haplotyped the simulated AIL data using several different values of the stringency criterion ranging from 0.5 to 1.

### Comparison with other methods

The results from our GA based haplotyping method were compared with those from two other approaches: a newly developed deterministic algorithm [7] and a commonly used likelihood based method (Merlin) [12]. We only compared the performance for the markers where the parental origin of the alleles cannot be inferred with certainty from the genotype of the relatives (parents/offspring). A marker genotype is correctly ordered when the parental origin of the alleles were reconstructed as they are in the simulated data set. We then compared for each method the proportion of uncertain genotypes that were correctly inferred, incorrectly inferred, and non-inferred. A genotype order is considered non-inferred if the risk of error associated with this genotype did not meet the stringency criterion. The haplotype error rate is then calculated as the number of incorrect ordered genotypes divided by the number of uncertain genotype orders.

## Results

### AIL dataset

**Figure 4:**
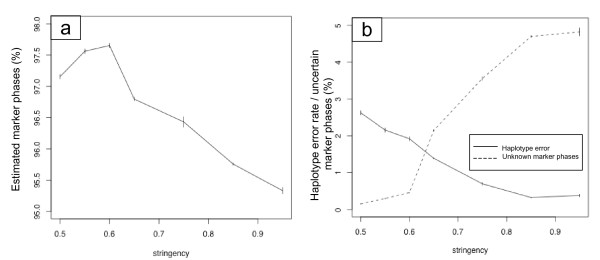
**Accuracy of the haplotyping algorithm as function of the stringency criterion**. a) Percentage of correct heterozygous marker phases in the estimated haplotypes. b) Percentage of haplotype error (plain dark line) and percentage of non estimated marker phases (dashed line).

To estimate the accuracy of the haplotyping method, we used the simulated AIL data described above. We report results from our method with different values of the stringency criterion in Figure 4. Increasing the stringency criterion from 0.5 to 1 decreased the power to infer haplotypes: the percentage of correctly ordered genotypes decreased from 97.1% to 95.1% (Figure 4a), whereas the accuracy increased from a haplotype error rate of 2.6% to 0.2% (Figure 4b). The proportion of uncertain haplotypes however increased from 0.15 to 4.8% (Figure 4b).

A deterministic method [7] was also used to infer the haplotypes in the AIL dataset. The results were comparable to those of a GA based method with stringency of about 0.75, with 96.7% correctly inferred phases and 3.3% haplotyping errors. (Table 1).

**Table 1:** haplotyping accuracy for the different compared methods

**Haplotyping method and data**	**correct haplotype (%)**	**haplotype** **errors (%)**	**unresolved** **cases (%)**
AIL pedigree			
GA- 0.95	95.10	0.20	4.80
GA- 0.5	97.10	2.60	0.15
Hapsim ^1^	96.70	3.30	0.00
Merlin ^2^	83.00	1.80	14.20
QTL-MAS pedigree			
GA- 0.95	87.00	0.05	13.00
GA- 0.5	90.00	1.00	9.00
Hapsim ^1^	97.00	3.00	0.00
Merlin ^2^	95.00	1.30	3.70

We compare three methods: our GA based method with high stringency (0.95), and low stringency (0.50), a deterministic method, and a likelihood based descent tree (Merlin), The results are given in percent of the non obvious heterozygous markers (non homozygous parents).

^1^Hernández-Sánchez and Knott (2009) [7]

^2^Abecasis et al (2002)[12]

When using the likelihood based method Merlin [12], the number of haplotype errors were low, at cost of a higher number of unresolved cases, with 83% correct haplotypes, 1.8% errors and 14.2% of unresolved cases. (Table 1)

### Dataset from 12^th ^QTLMAS workshop

We also inferred haplotypes in the publicly available dataset from the 12^th ^QTLMAS workshop [18]. Using a stringency criterion of 0.95, 87% of the markers were correctly ordered, 0.05% were incorrectly ordered and 13% remained unresolved. With a stringency of 0.5, the same method gave 90% correct haplotypes, 1% errors, and 9% unresolved cases. In comparison, the deterministic method [7] produced a higher rate of inferred phases with 97% of correct haplotypes, but also a higher error rate with 3% of haplotyping error. MERLIN [12] produced 95% of correct haplotypes, 1.3% of error, and 3.7% of unresolved cases (Table 1).

## Discussion

We propose a genetic algorithm (GA) based method for estimating multi-locus haplotypes in complex, multi-generational pedigrees, where the user simply can choose the stringency with which haplotypes are inferred. In our implementation, the user selects a stringency criterion as well as the number of iterations (k) used to estimate the stringency. The implemented stringency criterion is the minimum frequency of detection of the same optimum over *k *iterations for a marker to be considered as ordered. We have explored the performance of the algorithm for various levels of this stringency parameter and shown that it is possible for a user to change the properties of the inferred haplotypes to satisfy differences in the tolerance to haplotyping error or non inferred haplotypes. The choice of the parameter *k *depends on the structure and size of the data. The algorithm will, by definition, compute more reliable haplotypes with high values of k, but since the GA is the most time consuming step of the algorithm, using high values of *k *when analyzing large datasets considerably increases the computation time. Our tests indicate that stable results can be obtained using the arbitrary value of 100 for parameter *k *and that values as small as 30 give useful results.

The results obtained from our algorithm were compared to those obtained with two other methods: one deterministic based on MRHC [7], and Merlin: a likelihood based method [12]. For a simulated AIL data set, our method and the deterministic one provided similar results in terms of the proportion of correctly inferred marker phases. Merlin's results are as accurate as the ones reported by other methods regarding the small proportion of erroneous haplotypes they contain, but leaves a higher proportion of unresolved phases. We believe that this higher rate of unresolved cases is due to the fact that Merlin divides the data into full sib families, whereas the two other methods import and analyse the data as one single pedigree. The subdivision of the data is then expected to reduce the amount of information that can be utilized by the algorithm, especially with deep pedigree like the AIL where the distance between two related individuals can be more than two generations. Merlin is however expected to perform better in full sib based datasets.

We chose to compare the results of our method with those of Merlin for its reliability, and ease to use on different computer platforms. Moreover, its descent tree likelihood based approach represent an alternative between the purely deterministic and the genetic algorithm approach.

When comparing the results of the three different approaches on the 2008 QTL MAS data [18], we observe a lower proportion of inferred phases from our method, which was expected as we used a very high stringency parameter. Decreasing the stringency increased the amount of inferred haplotypes, but the power and error rate of our GA based method was still lower than the one reported by the two other methods, which indicates that for such data, our approach might be more conservative.

## Conclusion

We propose a general method to reconstruct haplotypes from pedigree and genotype data in complex pedigrees. Our introduction of a stringency criterion allows the user to control the rate of haplotype errors by discarding the ordered genotypes associated with higher risk of error. The method also provides an estimation of the error risk associated to each ordered genotypes, and allows the user to select how stringent one wants to be in reconstructing genotype order. As in [12], some genotypes might still be unresolved after the haplotyping procedure is completed if the data does not allow for haplotype assignment.

When compared with two existing methods utilizing different haplotyping strategies our method performs similarly well for inferring the parental origin of the alleles in complex pedigrees.

The stringency criterion allows the user to better control the error rate: a high stringency will produce haplotyped data with very few haplotype errors but a higher number of unordered genotypes. This configuration is suitable for the analysis of deep pedigrees like the nine generations AIL, and when the following steps of the analysis can handle data that contain a mixture of known and unknown marker phases. A low stringency will produce haplotyped data that contains few or no unordered genotypes, but an increased amount of haplotype errors. This configuration is suitable when haplotype errors can be tolerated, and when further analyses require all genotype order to be assigned. We believe that it is important to provide such a choice, as there will be different requirements imposed by analyses performed using the haplotyped data regarding the tolerance for haplotype errors or uncertain genotype order.

## Authors' contributions

ÖC initiated the study. ÖC and FB designed and developed the project and wrote the paper together. All authors have read and approved the manuscript.
